# Evaluation of facility and community-based active household tuberculosis contact investigation in Ethiopia: a cross-sectional study

**DOI:** 10.1186/s12913-019-4074-5

**Published:** 2019-04-22

**Authors:** Fana Tefera, Gena Barnabee, Anjali Sharma, Beniam Feleke, Daniel Atnafu, Negasi Haymanot, Gabrielle O’Malley, Getachew Feleke

**Affiliations:** 1 University of Washington, International Training and Education Center for Health (I-TECH), Seattle, WA USA; 2 Centers for Disease Control and Prevention- Ethiopia (CDC-Ethiopia), US Embassy, Entoto Road, P.O. Box 19284, Addis Ababa, Ethiopia; 3 Tigray Regional Health Bureau, Mekelle, Ethiopia; 4 International Training and Education Center for Health (I-TECH Ethiopia), Addis Ababa, Ethiopia

**Keywords:** Yield, Active case finding, Health extension workers, Implementation

## Abstract

**Background:**

No established strategy for household tuberculosis (TB) contact investigation (HTCI) exists in Ethiopia. We implemented integrated, active HTCI model into two hospitals and surrounding community health services to determine yield of active HTCI of all forms of TB and explore factors associated with active TB diagnosis in household contacts (HHCs).

**Methods:**

Case managers obtained HHC information from index cases at TB/DOTS clinic and liaised with health extension workers (HEWs) who screened HHCs for TB at household and referred contacts under five and presumptive cases for diagnostic investigation.

**Results:**

From 363 all forms TB index cases, 1509 (99%) HHCs were screened and 809 (54%) referred, yielding 19 (1.3%) all forms TB cases. HTCI of sputum smear-positive pulmonary TB (SS + PTB) index cases produced yield of 4.3%. HHCs with active TB were more likely to be malnourished (OR: 3.39, 95%CI: 1.19–9.64), live in households with SS + PTB index case (OR: 7.43, 95%CI: 1.64–33.73) or TB history (OR: 4.18, 95%CI: 1.51–11.55).

**Conclusion:**

Active HTCI of all forms of TB cases produced comparable or higher yield than reported elsewhere. HTCI contributes to improved and timely case detection of Tuberculosis among population who may not seek health care due to minimal symptoms or access issues. Active HTCI can successfully be implemented through integrated approach with existing community TB programs for better coordination and efficiency. Referral criteria should include factors significantly associated with active disease.

## Background

Ethiopia is among 30 post-2015 high-burden countries for tuberculosis (TB) [1]. National prevalence rates have halved since 1990 to an estimated 200/100,000 population in 2014 [1]. However, in 2010–2014, case detection rates decreased from 66 to 60% [1]. Lack of routine access to better diagnostics such as Xpert MTB/RIF and culture as well as quality control contribute to low detection rates [1–4]. Lack of access to and coordination across TB services and facilities delays case detection and treatment, increasing transmission risk [5–9].

The World Health Organization (WHO) [10] and STOP TB Strategy [11] recommend contact investigation to increase case finding. The yield of new active TB cases is greater among household contacts (HHCs) than the general population [12, 13]. Recent meta-analyses of household TB contact investigation (HTCI) in low- and middle-income countries revealed a prevalence of 3.1% all forms TB [12] and 1.5% bacteriologically confirmed TB [14] among HHCs. However, implementation in low-income countries remains limited with significant heterogeneity in policies, procedures, and results [12–15]. In Ethiopia, no established implementation strategies exist for HTCI [16]. Recent studies of HTCI in routine settings have focused on passive investigation [17–21]. A 2013 study in southern Ethiopia [22] utilizing health extension workers (HEWs) to actively screen and collect sputum from symptomatic HHCs at home yielded 5.3% SS + PTB cases among those tested; however, training, laboratory, and supervision components may impact feasibility of scale-up.

This paper presents results from a study of active HTCI integrated into routine facility- and community-based TB services in northern Ethiopia. The purpose of the study was to determine HTCI yield from two high TB caseload hospitals, compare findings across three operational index case definitions, and explore factors associated with active TB in HHCs to inform policies and procedures necessary to facilitate scaled active HTCI implementation in Ethiopia.

## Methods

This cross-sectional study evaluates an integrated model of active HTCI implemented across facility-based TB/DOTS services and the community-based 1HEW programme in northern Ethiopia. Study sites were Felegehiwot Referral and Mekelle hospitals, two high TB caseload public hospitals in Amhara and Tigray regions, respectively. The study population included patients with new, relapse, retreatment, and return after default all forms TB living in 34 woredas (districts) located within 100 km radius of each study hospitals, and their HHCs. We excluded multi-drug resistant (MDR) cases given lack of routine access to MDR-TB diagnostic services. No additional selection criteria were applied. Study period lasted from February 04, 2015 to July 28, 2015 for 5 months.

### Terms and definitions

Index case: first TB patient identified in the household registered at a TB/DOTS clinic, with at least one HHC, not necessarily source cases. Four operational definitions specify form of TB: all forms TB, Clinically diagnosed Pulmonary Tuberculosis (Clinically diagnosed PTB), Extra Pulmonary TB (EPTB) and SS + PTB.

Household contact (HHC): person living in the same house with an index case at registration and have lived for at least seven consecutive days in a month within the past 3 months and, not on TB treatment.

Malnutrition was assessed using a WHO adopted standard mean arm circumference (MUAC) tape with age-appropriate classification of nutritional status.

Tuberculosis case definitions align with WHO recommendations [1].

Diagnosis of active TB disease followed national guidelines [16]. PTB was diagnosed by sputum smear positive (SS+) result for acid-fast bacilli (AFB) examined using Ziehl-Neelsen or fluorescent sputum smear microscopy (SSM) or, sputum smear negative (SS-) or no smear with abnormal chest x-ray (CXR) at clinician discretion. Xpert MTB/RIF and culture are not routine diagnostics and were performed only at clinician discretion. Extrapulmonary TB (EPTB) was diagnosed by suggestive finding from histology, radiology/imaging or other body fluid analyses at clinician discretion. In contacts less than five, TB was diagnosed based on clinical signs and symptoms and abnormal CXR or suggestive tissue/fluid analysis. Tuberculin test was not performed, as it was not part of the national guideline.

Yield: proportion screened HHCs newly diagnosed with all forms active TB.

Number needed to be screened (NNS): Number of HHCs needed to be screened to detect one active TB case, calculated as 100/% yield [14, 23].

### Integrated active HTCI model

**Fig. 1:**
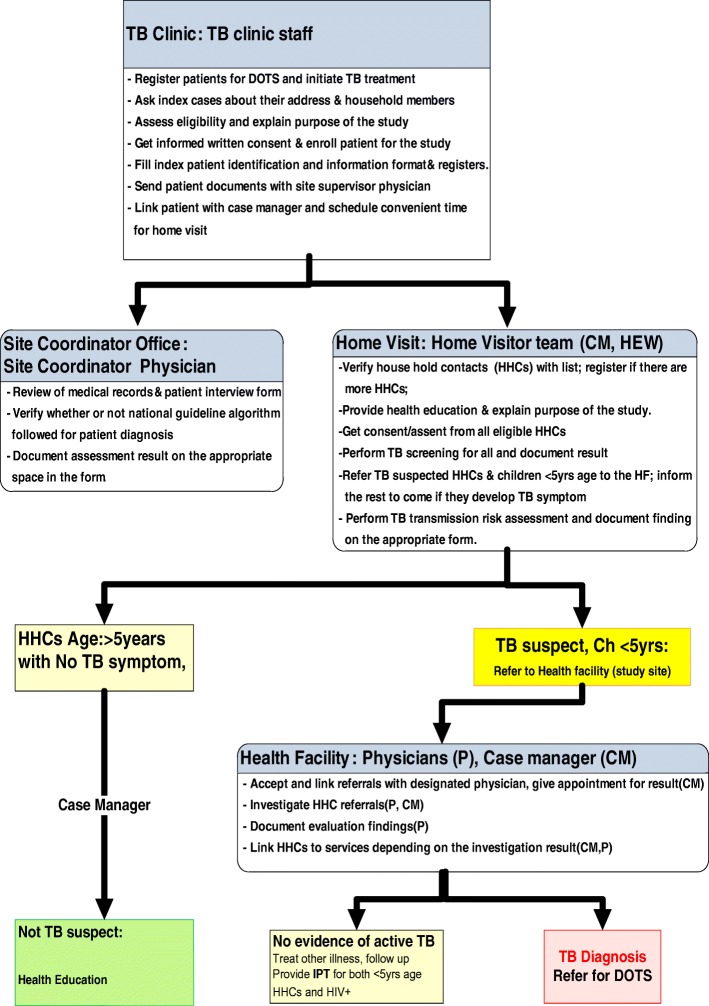
Participant enrollment and contact investigation workflow

Our model aligns with WHO HTCI recommendations [10]. TB focal persons in study hospitals enrolled eligible TB patients as index cases. Trained HTCI case managers recorded household location and number, age, and sex of HHCs. They provided information to Woreda/city TB focal persons and coordinated with HEWs to conduct household visits. At the household, HEWs enrolled and screened eligible HHCs using a TB screening tool adapted from WHO [24] and referred presumptive TB cases (one or more symptoms) and all under-five contacts to the study hospital for clinical investigation. If necessary, HEWs conducted one additional screening visit. They asked referred HHCs to present for investigation at the study hospital within 2 weeks and reminded those not presenting in time by phone (Please see Fig. 1). A physician conducted clinical examination, diagnostic testing, diagnosis and TB management including TB preventive therapy (TPT) as per the national guidelines [16].

The study provided phone cards and, if appropriate, travel reimbursement per completed household visit for the case manager, TB focal person and HEW and, reimbursement for transportation, per diem, and accommodation costs for referred HHCs and one parent/guardian.

### Data collection and analysis

Clinical data for index cases and clinically investigated HHCs were abstracted from patient files. We collected sociodemographic and risk factor data per participant and, household conditions data per household using piloted structured questionnaires in local languages. Data was entered into Epi Info (Version 7.1.5, CDC: Atlanta, GA, USA) in English. Data quality assurance methods included routine randomized review of electronic data against paper-based forms and validity checks between related variables across the electronic database. Co-investigators trained study and hospital staff involved in HTCI implementation and data collection and management. While HEWs have basic training in TB screening and prevention, trained HTCI case manager accompanied each HEW at least once to train and supervise data collection.

Data were extracted and analyzed using IBM SPSS Statistics (Version 19.0, IBM Corp) and Stata (Release 12, StataCorp LP). We analyzed HTCI flow, yield and NNS by region and operational index case definition. Yields are reported as proportions and per 100,000 with 95% confidence intervals (CI) using exact binomial method.

We described characteristics of index cases and HHCs using frequencies. We explored factors associated with active TB among HHCs using a multivariate generalized estimating equation (GEE) model composed of known active TB [8] risk factors found significant at *p* < 0.1 in bivariate analysis. Results are reported as odds ratios (OR) with 95% CI, statistically significant at *p* < 0.05.

### Ethical considerations

The University of Washington, Ethiopian National Ethics Review Committee, and U.S. Centers for Disease Control and Prevention granted ethical approval. Per Ethiopian guidelines, written informed consent for all persons 18 and older, parental consent for all children under 18, and assent for children 12–17 years old were obtained before enrolment. Household visits began with permission of household head or equivalent. Persons refusing participation were advised to visit the nearest facility for TB screening.

## Results

Table 1 details HTCI flow, yield and NNS by index case definition. In total, 363 index cases were enrolled. Of 1528 HHCs identified, 1509 (99%) were screened at first (97%) or second (3%) household visits. The total time needed to complete these HHCI for the 1509 HHCs of 363 index cases was 5 months. Of these, 809 (54%) were referred to study hospitals for further clinical investigation. Of referred HHCs, 763 (94%) presented for investigation where 598 (78%) received diagnostic testing. Nineteen HHCs were diagnosed with active TB with overall yield of 1.3% and 79 NNS. Yields were highest and NNS lowest with HTCI of SS + PTB index cases (yield = 4.3%; NNS = 23) compared to HTCI of EPTB Index cases (yield = 1.2%; NNS = 85), and all forms of TB index cases (yield = 1.3%; NNS = 79). Clinically diagnosed PTB has the lowest yield (yield = 0.3%; NNS = 348.)

**Table 1:** Yield of household TB contact investigation by TB diagnosis of index

	All Forms TB	EPTB	Clinically Dx PTB	SS+ PTB
Index cases	363	232	88	43
HHCs identified	1528	1031	357	140
HHCs screened^a^ (% of identified)	1509 (99)	1021 (99)	348 (97)	140 (10)
Referred^a^ (% of screened)	809 (54)	594 (58)	154 (44)	61 (44)
*< 5, no symptoms (% of referred)*	78 (10)	60 (10)	17 (11)	1 (2)
*Symptoms (% of referred)*	731 (90)	534 (90)	137 (89)	60 (98)
Investigated^a^ (% of referred)	763 (94)	557 (94)	151 (98)	55 (90)
Tested^a^ (% of investigated)	598 (78)	425 (76)	125 (83)	48 (87)
*SSM*^*b*^*(% of tested)*	175 (29)	100 (24)	52 (42)	23 (48)
*GeneXpert*^*b*^*(% of tested)*	7 (1)	7 (1)	0 (0)	0 (0)
*Other*^*c*^*(% of tested)*	416 (70)	318 (75)	73 (58)	25 (52)
Diagnosed^a^ (% Yield^d^)	19 (1.3)	12 (1.2)	1 (0.3)	6 (4.3)
Yield per 100,000 (95% CI^e^)	1259 (759–1959)	1175 (609–2044)	287 (7–1591)	4286 (1589–9095)
NNS (95% CI)	79 (55–132)	85 (49–164)	348 (63–1375)	23 (11–63)

*Abbreviations*: *TB* tuberculosis, *EPTB* Extra pulmonary TB, *PTB* Pulmonary TB, *DX* Diagnosed, *SS+* sputum smear positive, *SSM* sputum smear microscopy, *NNS* number needed to screen to detect one TB case

^a^Presented as n (%) unless otherwise indicated

^b^Indicated test ± any test indicated in the subsequent rows

^c^Other tests included histopathology (FNAc), imaging (CXR, ultrasound), or other body fluid analysis

^d^Percent (%) yield calculated as number diagnosed with TB divided by the number screened

^e^Yield per 100,000 with 95% confidence interval using exact binomial method

Females represented 51% of index cases and 50% of HHCs. 90% of index cases and 52% of their HHCs were aged 15 years and older (See Table 2). Moreover, 8% of index cases were HIV positive and 12% had SS + TB. Among HHCs, 56% shared bed with index cases and 96% were exposed to the index case for more than 31 days (See Table 2).

**Table 2:** Demographic, clinical, and exposure characteristics of index cases and household TB contacts

Characteristics	Index Cases		Household TB contacts	
	*N* = 363	%	*N* = 1509	%
Sex				
Male	179	49%	750	50%
Female	184	51%	759	50%
Age group				
0–4	2	1%	192	13%
5–14	33	9%	535	35%
15+	328	90%	782	52%
HIV status^a^				
HIV-negative	275	76%	1503	99.6%
HIV-positive	31	8%	6	0.4%
Unknown status	57	16%	–	–
Cough in index case				
No	199	54%	868	57.5%
Yes	167	46%	641	42.5%
TB diagnosis in index case				
EPTB	232	64%	1021	68%
Clinically Dx PTB	88	24%	348	23%
SS+ PTB	43	12%	140	9%
Relationship to index case				
Other	–	–	611	40%
Offspring/spouse	–	–	898	60%
Sleep area of index case				
Alone in separate rm.	–	–	184	12%
Shared bed	–	–	847	56%
Separate bed, shared rm.	–	–	478	32%
Duration of exposure				
7–30 days	–	–	45	4%
31+ days	–	–	1455	96%

*Abbreviations*: *TB* tuberculosis, *EPTB* extra pulmonary tuberculosis, *Dx* diagnosed, *SS+* sputum spear positive, *PTB* pulmonary tuberculosis, *rm* room

^a^Household TB contact HIV status is reported by self or parent/guardian at household visit

Factor analysis (Table 3) revealed HHCs with active TB were more likely to be malnourished (OR: 3.39, 95%CI 1.19–9.64), and live in households with a SS + PTB index case (OR: 7.43, 95%CI 1.64–33.73) and history of active TB (OR:4.18, 95%CI 1.51–11.55). While statistically significant in bivariate analysis, having an immunodeficiency condition, relationship to index case and sleeping in the same room with livestock were not statistically significant in the multivariate model. Age, sex, marital status, sleeping area of index case and crowding were not significant in bivariate analysis (data not shown). Data were insufficient to analyze HIV status and ventilation.

**Table 3:** Factors associated with active TB diagnosis among HHCs in Amhara^a^

	No TB	TB	OR (CI)^b^	Adjusted OR (CI)^c^
Host risk factors				
Nutritional status				
No malnutrition	1083 (73)	8 (42)	1.0 (ref)	1.0 (ref)
Malnutrition	404 (27)	11 (58)	**3.69 (1.19–11.44)**	**3.39 (1.19–9.64)**
Immuno-deficiency^d^				
Absent	1450 (97)	16 (84)	1.0 (ref)	1.0 (ref)
Present	40 (3)	3 (16)	**6.80 (1.90–24.34)**	3.70 (0.86–15.87)
Environmental risk factors				
TB diagnosis in index case				
EPTB	1009 (68)	12 (63)	1.0 (ref)	1.0 (ref)
SS+ TB	134 (9)	6 (32)	**3.77 (1.06–13.34)**	**7.43 (1.64–33.73)**
Clinically Dx TB	347 (23)	1 (5)	0.24 (0.03–1.87)	0.43 (0.05–3.68)
HHC relationship to index case				
Other	598 (40)	13 (68)	1.0 (ref)	1.0 (ref)
Offspring/spouse	892 (60)	6 (32)	**0.31 (0.11–0.89)**	0.39 (0.15–1.02)
Household sleeps in same room as livestock				
No	1085 (73)	9 (47)	1.0 (ref)	1.0 (ref)
Yes	405 (27)	10 (53)	**2.98 (1.05–8.48)**	3.36 (0.92–12.28)
TB history in household				
No	1336 (90)	12 (63)	1.0 (ref)	1.0 (ref)
Yes	154 (10)	7 (37)	**5.06 (1.77–14.44)**	**4.18 (1.51–11.55)**

*Abbreviations*: *TB* tuberculosis, *EPTB* extra-pulmonary TB, *SS+ PTB* sputum smear positive pulmonary TB, *Dx* diagnosed

^a^Presented as n (%) unless otherwise indicated

^b^Bivariate GEE analysis, bold indicates statistical significance at *p* < 0.1

^c^Multivariate GEE analysis, bold indicates statistical significance at *p* < 0.05

^d^Self-reported; includes HIV, severe kidney disease, diabetes, previous or current cancer treatment, previous TB disease, and symptoms/signs of primary immunodeficiency (e.g., recurrent or chronic infections)

## Discussion

Our study shows that integrated active HTCI can achieve high rates of screening and referral completion to yield new TB cases in two regions of northern Ethiopia. Consistent with other studies, results demonstrate that HEWs can successfully contribute to active case finding by screening HHCs and referring presumptive cases, and improve access to TB services by screening and referring at the household-level [22, 25–27]. This study contributes to evidence recommending HTCI for SS + PTB index cases to yield the highest proportion of newly diagnosed TB cases.

Yield comparison across studies is challenging given wide variation in index case, HHC and yield definitions. In this study (Table 1), HTCI of SS + PTB index cases generated a higher yield of all forms TB cases among screened HHCs (4.3%: 4286 per 100,000) than found in recent meta-analyses in low-income, high TB-burden countries (3.1% [12], 1.8% [14]), active HTCI studies in Ethiopia (2.5% [17], 0.9% [22]) and estimated national TB prevalence (200 per 100,000). When considering only bacteriologically confirmed TB cases among screened HHCs, it yielded 1.4% (data not shown), which is comparable to recent studies (1.2% [12], 1.5% [14], 0.76% [17], 0.8% [22]). In addition, HTCI of all forms of TB index cases yielded (1.3%; 1259 per 100,000) cases of TB and HTCI of EPTB index cases yielded (1.2%; 1175 per 100,000) cases (See Table 1), a six-fold increase compared to the general population prevalence. This supports the definition in our and other studies of index cases as the first patient identified within the household to initiate HTCI and not necessarily being source cases [28]. Studies of passive HTCI have observed higher yields of all forms TB cases among HHCs receiving facility-based screening or testing (6.5% from PTB index cases [19]; 10.1% from all forms index cases [18]) possibly due to appropriate self-referral. HTCI of clinically diagnosed PTB index cases generated a lower yield of all forms TB cases among screened HHCs (yield = 0.3%; 287 per 100,000) (Table 1). This could be due to use of clinical criteria (sign and symptom complex, non-response to antibiotic regimen, CXR, low quality smear etc.) to make clinical diagnosis of PTB may result in incorrect TB diagnosis thus contact investigation may not have been needed in the first place. However further study may be needed using multiple supportive diagnostic techniques with better specificity (such as imaging techniques) for establishing firm diagnosis in the absence of bacterial confirmation before concluding that these group are indeed low yield groups.

Factor analysis results (Table 3) suggest a more sensitive screening tool for the Ethiopian context could include household history of TB, malnutrition, and possibly immunodeficiency signs/conditions as referral criteria. Replicable, low-cost design elements including a dedicated HTCI case manager and household screening and follow-up via HEWs [22]; use of trusted persons in the community speaking the local language [10]; provision of referral slips [29]; household-specific TB education [19]; and a dedicated process for receiving and investigating referred HHCs at facilities to reduce opportunity costs [30–32] may have contributed to the model’s high screening and referral completion rates. Notably, this model introduced HTCI case manager as the only new cadre to the workforce. The 16 health packages HEWs address includes TB; however, workload is a concern given competing priorities and shortages in rural areas [33]. Reimbursement of transportation and diagnostic costs likely increased referral completion [31], which may still be achieved without reimbursement if investigation is decentralized. Health centers provide sputum examination at no-cost. Lack of chest x-ray and other more sensitive diagnostics may reduce yield particularly if HHCs cannot travel to hospitals for further investigation.

Given people recently infected with *Mycobacterium tuberculosis* are at increased risk for the development of active TB within 1–2 years after the acquisition of the infection [28], HTCI guidance should consider timing for screening, inclusion of risk-based referral criteria, and follow-up of asymptomatic HHCs. Disparate rates of diagnostic testing performed suggest that clinical screening algorithms and diagnostic guidelines specific to HTCI are necessary to guide routine implementation. While the least restrictive criteria (i.e., diagnostic testing for all contacts) is associated with increased yield [14, 34], guidelines must consider cost to the health system as this strategy may not be as effective in preventing transmission.

This study has several limitations. While consistent with operational research, non- randomization limits generalization of findings. Our small sample size reduces precision of point estimates and may be insufficient in detecting any but the largest statistical differences. Difficulty of TB diagnosis in the Ethiopian context, particularly in early stages of manifestation [35], could have resulted in undiagnosed cases in both regions. Conversely, TB may be clinically over-diagnosed including the eight cases with pleural fluid and FNAC results suggestive of TB, in the absence of routine access to Xpert MTF/RIF and culture for TB for both index cases and HHCs (Table 4). High proportion of EPTB cases observed in this study requires further investigation. While we cannot establish the contribution of our model to overall case notification in the region, relative contribution to notification is associated with increased index case coverage [14] for which we achieved 98% in this study. Financial and temporal constraints prevented cost analyses, sensitivity analyses, and comparison with passive and other models of active HTCI.

**Table 4:** Age, sex, health status and clinical details of HHCs diagnosed with active TB disease

HHC	Age	Sex	Symptoms at screening	TB Dx in index case	Mal-nutrition	Immuno-deficiency^d^	Active TB diagnosis in HHCs^e^	TB Site(s)	Diagnostic results^f^
Amhara Region									
1	58	M	Cough, night sweats, weight loss, appetite loss, fatigue	SS + PTB	No	No	EPTB	Cervical	AFB-negative SSM, normal CXR, suggestive FNAc
2	53	M	Cough, night sweats, fatigue	EPTB	No	No	EPTB	Pleural	Abnormal CXR, suggestive body fluid analysis
3	2	M	Fever, appetite loss	SS + PTB	Yes	No	PTB, no SSM	Pulmonary	Abnormal CXR
4^a^	11	M	Fever, night sweats, weight loss, appetite loss	SS + PTB	No	No	PTB, no SSM	Pulmonary	Abnormal CXR
5^a^	23	F	Cough, fever, night sweats, weight loss, appetite loss, fatigue	SS + PTB	No	No	SS + PTB	Pulmonary, cervical	AFB-positive SSM, abnormal CXR, suggestive FNAc
6^a^	40	F	Cough, fever, night sweats, weight loss, appetite loss, fatigue	SS + PTB	No	No	EPTB	Pleural	AFB-negative SSM, abnormal CXR, suggestive FNAc, suggestive U/S
7	14	F	Cough, neck swelling	EPTB	Yes	No	EPTB	Cervical	Suggestive FNAc
8	55	M	Cough, fever	SS-PTB	Yes	Yes	SS-PTB	Pulmonary	AFB-negative SSM, abnormal CXR
9	14	F	Cough, fever, night sweats, haemoptysis	EPTB	Yes	No	PTB, no SSM	Pulmonary	Abnormal CXR
10	5	M	Cough, fever, appetite loss	EPTB	No	No	PTB, no SSM	Pulmonary	Abnormal CXR
11	28	F	Fever, night sweats	EPTB	Yes	No	EPTB	Hilar lymph-adenopathy	Abnormal CXR
12	30	F	Cough, fever, fatigue	EPTB	Yes	Yes^c^	EPTB	Axillary lymph-adenopathy	Normal CXR, suggestive FNAc
13	8	M	Night sweats	EPTB	No	No	PTB, no SSM	Pulmonary, cervical	Abnormal CXR, suggestive FNAc
14^b^	60	F	Cough, fever, night sweats, weight loss, appetite loss, fatigue	EPTB	Yes	No	EPTB	Pleural, cervical	AFB-negative SSM, abnormal CXR, suggestive FNAc
15^b^	20	F	Cough, fever, night sweats	EPTB	Yes	Yes	PTB, no SSM	Pulmonary	Abnormal CXR
16	10	M	Cough, fever, night sweats, appetite loss	EPTB	Yes	No	PTB, no SSM	Pulmonary	Abnormal CXR
17	15	F	Cough, fever, night sweats, weight loss, neck swelling, fatigue	EPTB	Yes	No	PTB, no SSM	Pulmonary	Abnormal CXR, suggestive FNAc
18	4	F	Fever, night sweats	EPTB	No	No	PTB, no SSM	Pulmonary	Abnormal CXR
Tigray Region									
19	14	F	Cough, weight loss	SS + PTB	Yes	No	SS + PTB	Pulmonary	AFB-positive SSM, abnormal CXR

^a^, ^b^ Observed clustering (HHCs within the same household)

^c^Only HHC diagnosed with active TB disease with a positive HIV status

^**d**^Self-reported; includes HIV, kidney disease, diabetes, previous/current cancer treatment, previous TB, signs of primary immunodeficiency (e.g., recurrent or chronic infections)

^e^*SS-PTB* Smear-negative pulmonary TB, *SS + PTB* smear-positive pulmonary TB, *PTB* pulmonary TB, *no SSM* sputum smear microscopy not performed, *EPTB* extra pulmonary TB

^f^*AFB* acid-fast bacilli, *SSM* sputum smear microscopy, *CXR* chest x-ray, *FNAc* fine needle aspiration cytology, *U/S* ultrasound

## Conclusion

This program evaluation finding demonstrated that active HTCI integrated into the existing health system produced high rates of HHC coverage, screening and referral completion. HHCI intervention from index cases with all forms of TB has a considerable high yield compared to the estimated prevalence in the general population. Therefore we recommend that HHCI should be done for all forms of TB index cases. However, where there is resource limitation priority should be given to HHCI of SS+ followed by EPTB index cases. It is also our recommendation that HTCI be done integrated with the existing community TB control where the structure exists. Standard protocols and procedures must be developed to improve efficiency and consistency. In addition, screening and investigation algorithms that include factors highly associated with increased risk of TB can further increase yield. Implementation must be coupled with efforts to improve passive case finding and access to rapid and sensitive diagnostic services. Routine monitoring and evaluation of HTCI is necessary and its contribution to case notification and cost-effectiveness should be further investigated.

## Abbreviations

AFB: Acid fast bacilli
CXR: Chest x-ray
EPTB: Extra pulmonary TB
GEES: Generalized estimating equation
HEWs: Health extension workers
HHC: Household contacts
HTCI: Household tuberculosis contact investigation
MUAC: Mean arm circumference
NNS: Number needed to be screened
SS-: Smear negative
SS+: Smear positive
SSM: Sputum smear microscopy
TB: Tuberculosis
TB/DOTS: Tuberculosis directly observed therapy short course
WHO: World Health Organization

## Footnotes

1 HEWs are women aged 18 years or older with at least 10th grade education who are nominated by the local community, representatives in which they reside in order to ensure acceptance by community members. They are government salaried community health workers and implementing The Extension Program (HEP) which is a defined package of basic and essential promotive, preventive and selected high impact curative health services including community TB program targeting households.
